# Transcriptome Analysis of the Late-Acting Self-Incompatibility Associated with RNase T2 Family in *Camellia oleifera*

**DOI:** 10.3390/plants12101932

**Published:** 2023-05-09

**Authors:** Chang Li, Mengqi Lu, Junqin Zhou, Sen Wang, Yi Long, Yan Xu, Xiaofeng Tan

**Affiliations:** 1 Key Laboratory of Cultivation and Protection for Non-Wood Forest Trees, Ministry of Education, Changsha 410004, China; lichang00052@163.com (C.L.); lu_mengqi@163.com (M.L.);; 2 Academy of Camellia Oil Tree, Central South University of Forestry and Technology, Changsha 410000, China; 3 The Belt and Road International Union Research Center for Tropical Arid Nonwood Forest in Hunan Province, Changsha 410000, China

**Keywords:** Camellia oil tree, late-acting self-incompatibility, transcriptome, RNase T2 family

## Abstract

The Camellia oil tree (*Camellia oleifera* Abel.) is an important nonwood forest species in China, and the majority of its cultivars are late-acting self-incompatibility (LSI) types. Although several studies have examined the mechanism of LSI, the process is quite complicated and unclear. In this study, pollen tube growth and fruit setting of two Camellia oil tree cultivars Huashuo (HS) and Huajin (HJ) were investigated after non and self-pollination, and transcriptomic analysis of the ovaries was performed 48 h after self-pollination to identify the potential genes implicated in the LSI of Camellia oil trees. The results showed that the fruit set of HS was significantly higher than that of HJ after self-pollination. Transcriptomic analysis revealed that plant hormone signal transduction, the phosphatidylinositol signaling system, ATP-binding cassette (ABC) transporters, reactive oxygen species (ROS) metabolism, and Ca^2+^ signaling were mainly contributed in the LSI of reaction of Camellia oil tree. Moreover, nine RNase T2 genes were identified from the transcriptome analysis, which also showed that CoRNase7 participated in the self-incompatibility reaction in HS. Based on phylogenetic analysis, CoRNase6 was closely related to S-RNase from coffee, and CoRNase7 and CoRNase8 were closely related to S-RNase from Camellia sinensis. The 9 RNase T2 genes successfully produced proteins in prokaryotes. Subcellular localization indicated that CoRNase1 and CoRNase5 were cytoplasmic proteins, while CoRNase7 was a plasma membrane protein. These results screened the main metabolic pathways closely related to LSI in Camellia oil tree, and SI signal transduction might be regulated by a large molecular regulatory network. The discovery of T2 RNases provided evidence that Camellia oil tree might be under RNase-based gametophytic self-incompatibility.

## 1. Introduction

Camellia oil tree (*Camellia oleifera*), a vital member of the Camellia genus in Theaceae, is one of the most important evergreen tree species in China [1,2]. Almost 2300 years have passed since this species was first planted [3]. Because its seeds can be used to produce high-quality edible oil and because this species is widely cultivated, the camellia oil tree has emerged as one of the world’s four essential woody safe to eat oil tree species [4,5]. Research shows that the unsaturated fatty acids in camellia oil account for more than 90% of the total seed oil, which is a high-quality edible oil widely known as “Oriental olive oil” [6,7]. Camellia oil also contains many active ingredients, such as triterpenes, vitamins, squalene, tocopherols, carotene, saponins, glycerol, and alcohol [8]. Large amounts of camellia oil consumption can decrease cholesterol and improve cardiovascular and cerebral performance [1]. In *C. oleifera* production, self-incompatibility (SI) has historically been a significant factor affecting *C. oleifera* output [9], and under natural conditions, the fruiting setting rate of *C. oleifera* is less than 5% [8]. SI can also cause high heterogeneity within chromosomes, which hinders whole-genome sequence assembly and the construction of genetic maps of *C. oleifera* [10,11]. Elucidating the molecular mechanism of SI is therefore important for the development of the camellia oil tree industry.

In flowering plants, SI is a genetically controlled process that inhibits self-fertilization and facilitates outcrossing, which is beneficial for maintaining genetic diversity and resisting adverse environmental conditions [12]. SI is a widespread mechanism; more than half of all angiosperm species—more than 70 families and 250 genera—undergo SI [13,14]. Regardless of the fact that SI has been frequently observed in angiosperms, the molecular mechanism is derived from a small sample of just five families [14]. In general, SI systems in flowering plants can be classified into three main classes: sporophytic self-incompatibility (SSI), gametophytic self-incompatibility (GSI) and late-acting self-incompatibility (LSI) [15]. In SSI, the SI phenotype is determined by the diploid genotype of its anthers [16]. SI is regulated by two main genes at the S locus: S-receptor kinase (SRK), which is specifically expressed in the stigma, and the S-locus cysteine-rich (SCR) protein, which is specifically expressed in pollen. SI occurs when any one of the S genotypes in pollen from the anthers is the same as the S genotype of the pistils. SSI has been widely studied in Brassicaceae. In GSI, the gametophytic haploid genotype determines the SI phenotype [16], and it is regulated by a locus with multiple alleles (S loci) of two paired factors named S-RNase genes and F-box genes, which are, respectively, the female and male determinants of SI specificity [17,18,19]. There are many studies on female SI factors, and the cDNA of S-RNase, which is a type of ribonuclease, has been extracted and cloned from pistils in many different species, such as *Pyrus serotina* Rehd, *Malus domestica*, *Citrus* and *Petunia hybrids* [20,21,22,23]. In Papaveraceae species, there is another GSI mechanism wherein a Ca^2+^ signaling cascade causes pollen tubes to undergo programmed cell death (PCD) [10].

LSI is categorized according to the location at which pollen tubes are inhibited, as pollen tubes resulting from self-pollination do not reach the ovary; LSI is different from GSI and SSI, which are based on genetic mechanisms [15]. LSI has been reported in species of many diverse families, including *Ipomopsis aggregata* (Polemoniaceae), *Ceiba speciosa* (Bombacoideae), *Spathodea campanulata* (Bignoniaceae) and *Camellia sinensis* (Theaceae) [14,24]. However, the genetic control of LSI has been investigated little and remains controversial [25]. Lipow demonstrated that LSI in *Asclepias exaltata* is determined by an individual locus with numerous alleles, whereas via transcriptome analysis, Zhang suggested that the LSI of *C. sinensis* may be regulated by gametophyte [10]. PCD in SI is complex and likely regulated by a large network. Recently, researchers have focused on screening non-S factors and downstream signaling pathways involved in SI. Liwei et al. demonstrated that apple S-RNase could cause the accumulation of inorganic pyrophosphatase (Ppi), which inhibited tRNA aminoacylation and resulted in a reduction in pollen growth [26]. Reactive oxygen species (ROS) may function as secondary signaling molecules that are essential for the growth of pollen tubes, and self-PrsS in poppy pollen tubes increased the ROS content in a calcium-dependent manner [27,28]. In apple, cytoplasmic Ca^2+^ concentrations and Ca^2+^ signaling cascades cause pollen tube tip development to be halted, downstream actin filament depolymerization (AFs), and PCD as a result [19]. It has also been reported that S-RNase activity in the pollen tube causes changes in phospholipases [29]. Because the male determinants of SI have not been determined, research on *C. oleifera* SI tends to be slow. A previous study indicated that *C. oleifera* presented the characteristics of LSI [8], however, the underlying molecular process is not yet clear. No self-compatible *C. oleifera* varieties have been found, and studies of self-incompatibility in *Camellia oleifera* are based on self-and cross-pollination. Few studies have focused on compatibility differences across varieties. Our study investigated differences in the inbreeding affinity of two *C. oleifera* cultivars, and used transcriptome analysis to reveal the main pathway involved in LSI-based non and self-pollination. We found a candidate S-RNase within the RNA sequencing (RNA-seq) data from the ovaries at 48 h after self-pollination. We also evaluated another 8 RNase T2 genes that were identified in the transcriptome and expressed in the ovaries. To identify more candidate S genes associated with LSI in *C. oleifera*, we cloned these 9 RNases, and sequence phylogenetic, gene structure, prokaryotic expression, and subcellular localization analyses were performed to distinguish S-RNases from other members of the RNase T2 family. The results should help elucidate the mechanism of LSI in Camellia oil trees, which will provide a theoretical foundation for the production and cultivation of *Camellia oleifera*.

## 2. Results

### 2.1. Determination of the Occurrence of SI and Fruit Set Evaluation

**Figure 1:**
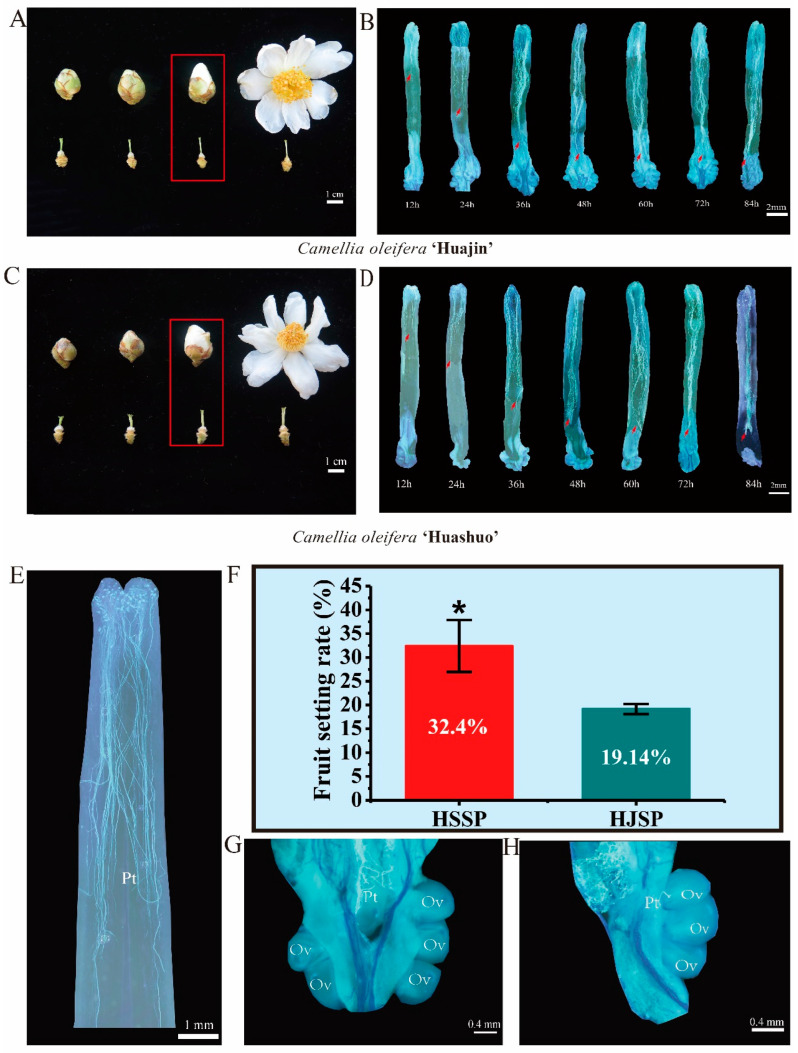
The growth of pollen tubes after 12 h to 84 h of self-pollination and fruiting setting rates in *Camellia oleifera* HJ and HS. (**A**) Flower bud at different developmental stages of HJ, Flower buds with red boxes are selected for pollination; (**B**) The growth of pollen tubes after 12 h to 84 h of self-pollination in HJ (40×); the red arrows indicate the length of the style reached by the pollen tubes (**C**) Flower bud at different development stages of HS; (**D**) The growth of pollen tubes from 12 h to 84 h of self-pollination in HS (40×); the red arrows indicate the length of the style reached by the pollen tubes (**E**) Normal growth pollen tube after self-pollinated in HS; (**F**) Fruit setting rate in Camellia oleifera HJ and HS (* *p* < 0.05); (**G**) Pollen tube grows to the ovary successfully 60 h after self-pollination in *Camellia oleifera* HJ. Pt = pollen tube, Ov = ovary. (**H**) Pollen tube grows to the ovary successfully 60 h after self-pollination in *Camellia oleifera* HS. Pt = pollen tube, Ov = ovary.

Flower buds at the large bud stage were selected for self-pollination (Figure 1A,C). Fluorescence microscopy observations demonstrated that pollen grains from both Huashuo (HS) and Huajin (HJ) germinated successfully after self-pollination, and the rate of elongation of the pollen tubes did not differ significantly from one another. The pollen tube of HJ was much faster than that of HS and had nearly reached the middle position of the style channel 24 h after self-pollination (Figure 1B,D). Both cultivars’ pollen tubes reached the bottom of the styles 48 h after self-pollination, but after that they grew more slowly and stopped, failing to reach the ovaries to complete double fertilization. However, 48 h after cross-pollination, the pollen tubes can be fertilized successfully [30]. These results are consistent with those of previous research [8]. As a result, we revealed that the SI of HJ and HS occurred between the style base and the upper ovary, and the SI occurred at 48 h after self-pollination. In addition, we found that a few pollen tubes could penetrate the ovary and reach the micropyle and complete double fertilization, which was similar to the processes involving cross-pollination (Figure 1G,H).

At two months after self-pollination, the fruit sets were investigated. We found that the fruit setting percentage of HS was 32.40%, which was significantly higher than that of HJ (19.14%) (Figure 1F). At the fruit ripening stage, the self-pollination fruit setting rate of *camellia oleifera* was less than 10%, and there were many factors affecting the fruit setting rates at this stage, which will not be discussed here. Overall, the observation and investigation indicate that *C. oleifera* is a late-acting self-incompatibility plant. There are also variations in the level of self-incompatibility in different *C. oleifera* varieties.

### 2.2. Transcriptome Assembly and Functional Annotation

The transcriptomes of HJSP and HSSP at 48 h after self-pollination were compared with those of HJNP and HSNP without pollination based on fluorescence microscopy observations of pollen tube growth. Twelve RNA-seq libraries were separately constructed by high-throughput sequencing. In total, 54.59~65.74 million reads of raw sequence data were obtained, and an average of approximately 58.90 million clean reads for each sample were obtained after being quality filtering. The Q30 was more than 94.01% for each sample, and the GC concentration varied from 43.08% to 43.39%. A total of 91.67% of the clean reads were successfully mapped to the reference genome of Camellia oil tree (Table 1). Approximately 15,091 unannotated unigenes were found (Supplementary Table S1), and 12,911 of these genes were annotated after blasting with four databases, with 12,911 unigenes in the NCBI nonredundant protein sequence (NR) database, 7462 in the Swiss-Prot Protein Database (Swiss-Prot), 6597 in the Gene Ontology (GO) database, and 2874 in the Kyoto Encyclopedia of Genes and Genomes (KEGG) database.

### 2.3. Comparative Analysis of Differentially Expressed Genes (DEGs)

**Figure 2:**
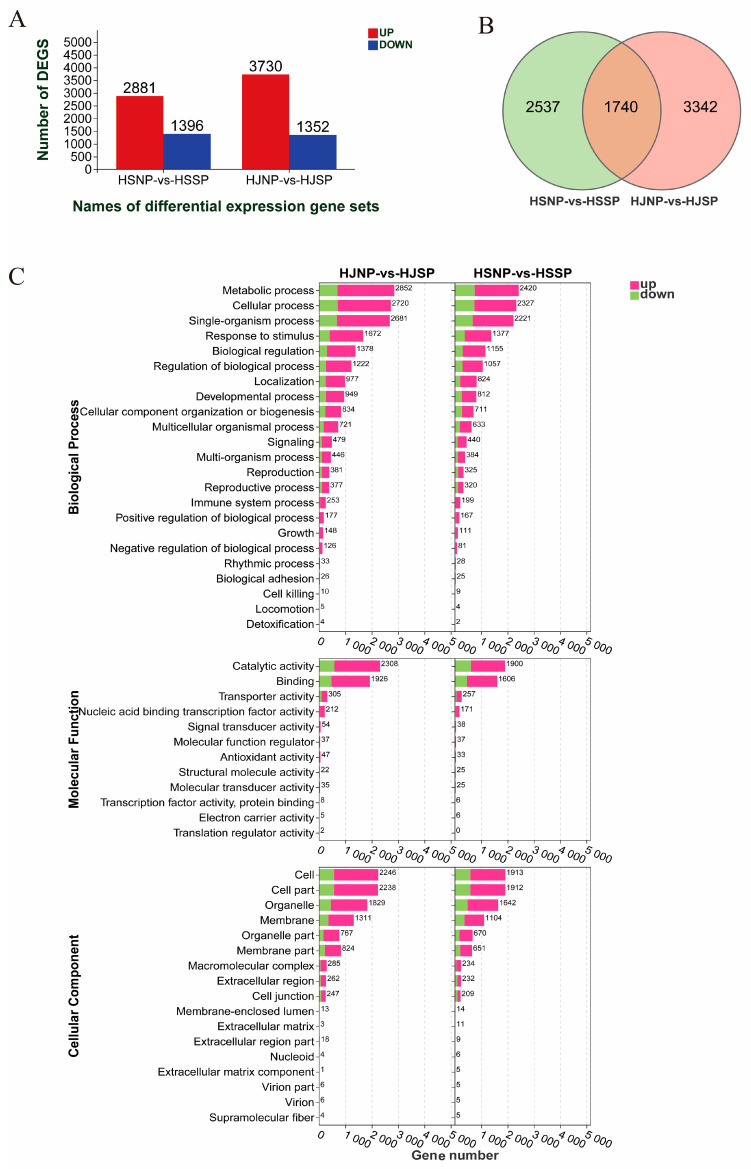
Analysis of DEGs in self- and nonpollinated *Camellia oleifera ovaries*. (**A**) Number of up- and downregulated DEGs in the two groups; (**B**) Venn diagram showing the number of DEGs in the two groups, the green circle represented DEGs in HSNP_HSSP comparison group, the pink circle represented DEGs in HJNP_HJSP comparison group; (**C**) GO function annotation of the ovary transcriptomes of the two cultivars *Camellia oleifera* ‘Huajin’ and ‘Huashuo’.

In this study, fragments per kilobase of transcript per million mapped reads (FPKM) were utilized to find the DEGs associated with the LSI of Camellia oil tree. In total, 5082 and 4277 DEGs were detected in the HJNP_HJSP and HSNP_HSSP comparisons, 3730 upregulated and 1352 downregulated genes were identified in the HJNP_HJSP comparison, and 2881 upregulated and 1396 downregulated genes were identified in the HSSP_HSNP comparison (Figure 2A), and 1740 common DEGs (1378 commonly upregulated and 339 commonly downregulated) were expressed in all the comparison groups (Figure 2B). These findings suggested that these 1740 DEGs play a crucial role in regulating the self-incompatibility response of *Camellia oleifera*.

### 2.4. GO and KEGG Pathway Analysis of DEGs

To assess whether the DEGs had any significant functional enrichment, GO term enrichment analyses were performed. Among them, the top three GO terms in the biological process category were ‘metabolic process’, ‘cellular process’ and ‘single-organism process’. The top three GO terms in the molecular function category were ‘catalytic activity’, ‘binding’ and ‘transporter activity’; and the top three GO terms in the cellular component category were ‘cell’, ‘cell part’ and, ‘organelle’ (Figure 2C).

**Figure 3:**
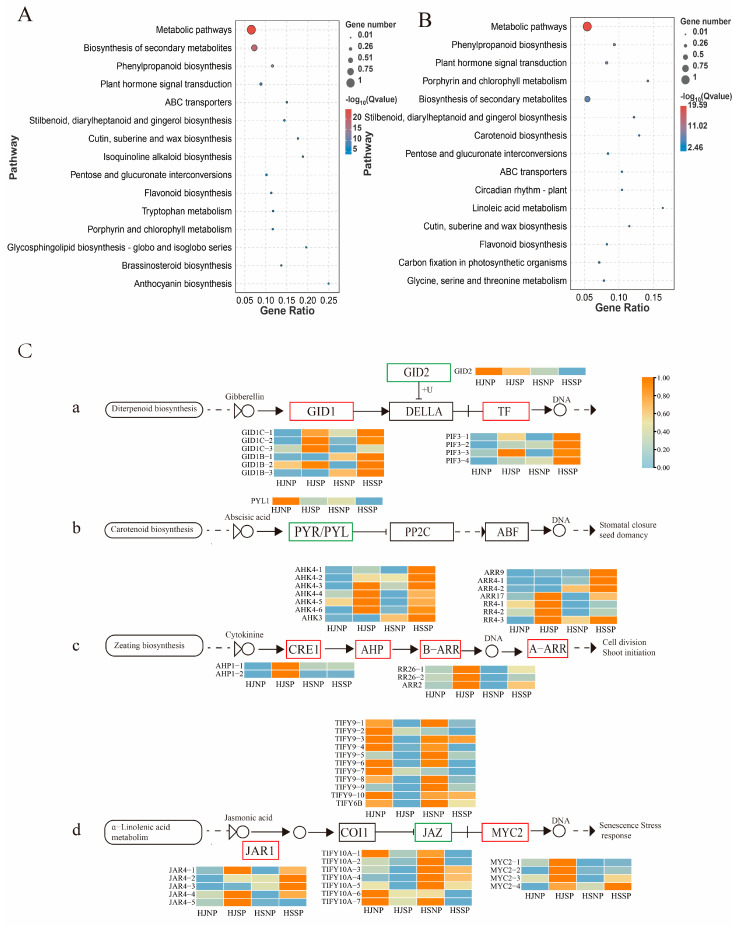
KEGG enrichment pathway analysis of DEGs in in self- and nonpollinated ovary of *Camellia oleifera.* (**A**) Annotation of the top 15 KEGG pathways of DEGs in HJNP_HJSP gene set. (**B**) Annotation of the top 15 KEGG pathways for DEGs in HSNP_HSSP gene set. (**C**) Genes with differential expression in hormone signaling pathways in the reaction to self-incompatibility in *Camellia oleifera* ‘Huajin’ and ‘Huashuo’. (**a**) The DEGs in the gibberellin signaling pathways in response to LSI in Camellia oil tree. (**b**) The DEGs in the abscisic acid signaling pathways in response to LSI in Camellia oil tree. (**c**) The DEGs in the cytokine signaling pathways in response to LSI in Camellia oil tree. (**d**) The DEGs in the jasmonic acid signaling pathways in response to LSI in Camellia oil tree.

According to KEGG pathway analysis, the HJNP_HJSP gene set and the HSNP_HSSP gene set each had 123 and 118 enriched pathways, respectively. All of the pathways from the two comparison groups could be grouped into five categories: metabolism, genetic information processing, environmental information processing, cellular process and organismal systems. The top 15 significantly enriched pathways of the two groups being compared are listed in Figure 3A,B. The major enriched pathways that might be related to SI were identified in both the HJNP_HJSP gene set and the HSNP_HSSP gene set: plant hormone signal transduction (ko04075), ATP-binding cassette (ABC) transporters (ko02010), phosphatidylinositol signaling system (ko04070), MAPK signaling pathway-plant (ko04016), plant–pathogen interaction (ko04626), flavonoid biosynthesis (ko00941), and ubiquitin-mediated proteolysis (ko04120). Among them, plant hormone signal transduction (ko04075), ABC transporters (ko02010), biosynthesis of secondary metabolites (ko01110), and phenylpropanoid biosynthesis (ko00940) were among the top 10 significantly enriched pathways (Figure 3A,B).

### 2.5. DEGs Related to Plant Hormone Signal Transduction

Plant hormone signals are involved in regulating plant physiological and biochemical processes to a large extent, including pollen–stigma interactions, seed germination and development, and pollen tube polar growth and so forth [31,32,33]. In this study, 102 DEGs and 112 DEGs were found in the gene sets HJNP_HJSP and HSNP_HSSP, respectively. Fifty-four of these DEGs, including several engaged in cytokine (CK), abscisic acid (ABA), gibberellin (GA), and jasmonic acid (JA) pathways were substantially expressed in the two comparison groups. The histidine-containing phosphotransfer protein gene (*AHP*), the two-component response regulator ARR-B family (ARR-B) and the two-component response regulator ARR-A family (ARR-A) were upregulated in the CK pathway of both groups. *AHP* was also upregulated in the HJNP_HJSP gene set, while there did not show any discernible differences in the HSNP_HSSP gene set. In the ABA signaling pathway, the ABA receptor PYL1-like gene (*PYL1*) was downregulated in the HJNP_HJSP gene set and was not significantly variable in the HSNP_HJSP gene set. In the GA signaling pathway, the phytochrome-interacting factor 3 gene (*PIF3*) and the gibberellin receptor GID1 gene (GID1) were upregulated in the two comparison groups, and GID2 was downregulated in HJSP compared to HJNP. The transcription factors MYC2-like (MYC2) and jasmonic acid-amino synthetase (JAR1) were upregulated in the JA signaling pathway of both comparison groups (Figure 3C), and JAZ, a protein with the jasmonate ZIM domain, was downregulated in both comparison groups. Taken together, these findings suggested that the LSI of Camellia oil trees may be significantly influenced by plant hormone signal transduction pathways.

### 2.6. DEGs Involved in ABC Transporters

**Figure 4:**
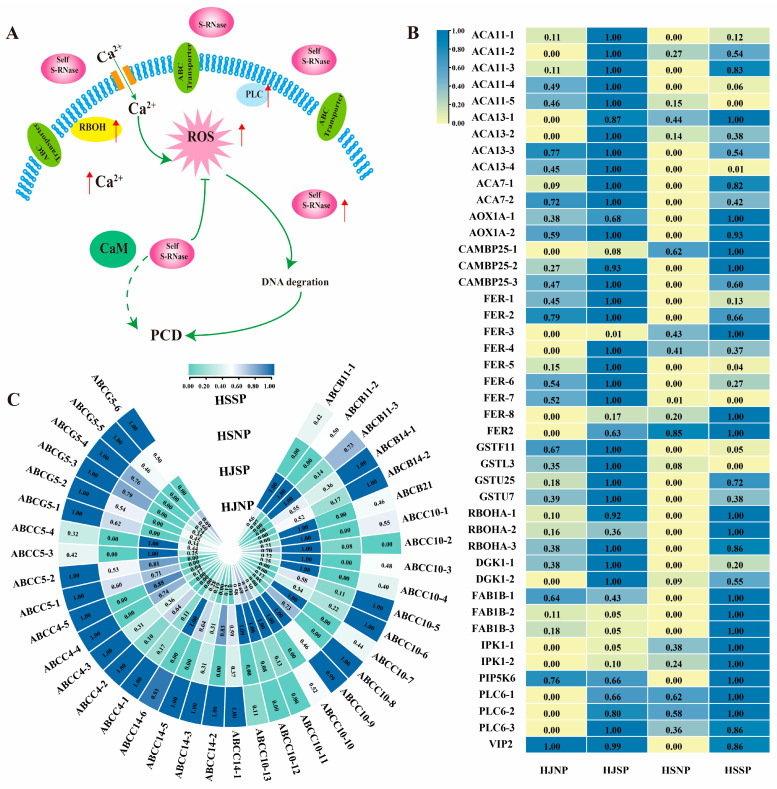
Expression profiles of genes in ABC transporters, the phosphatidylinositol signaling system, ROS and the Ca^2+^ signaling pathway. (**A**) Simplified illustration of most relevant molecule discovered to be related to LSI. (**B**) Comparison of expression patterns of vital genes related to phosphatidylinositol signaling system, ROS and Ca^2+^ signaling pathway in self- and nonpollinated ovaries. yellow and blue represent relative decreases and increases in expression, respectively (log_2_ fold change). The annotation of these genes is displayed in Supplementary Table S2. *ACA*: Calcium-transporting ATPase; CAMBP25: calmodulin-binding protein 25; *FER*: receptor-like protein kinase FERONIA; *GSTF*: glutathione S-transferase; RBOHA: respiratory burst oxidase protein like; *DGK*: diacylglycerol kinase 1-like isoform X2; *FAB1B*: 1-phosphatidylinositol-3-phosphate 5-kinase FAB1B-like isoform X1; *IPK*: inositol-pentakisphosphate 2-kinase; *PIP5K6*: phosphatidylinositol 4-phosphate 5-kinase 6-like; *PLC6*: phosphoinositide phospholipase C 6-like; VIP: nositol hexakisphosphate and diphosphoinositol-pentakisphosphate kinase VIP2-like. (**C**) Comparison of the expression profiles of important genes related to the ABC transporter pathway in self- and nonpollinated ovaries of *Camellia oleifera*. green and blue indicate relative decreases and increases in expression (log_2_ fold change). A list of these genes’ annotations may be found in Supplementary Table S3.

ABC transporter proteins compose a large class of transmembrane proteins that transport substrates across the lipid bilayer of the cell membrane mainly through the energy produced by the hydrolysis of ATP. It has been demonstrated that the transport of S-RNase was mediated by ABCF [34]. In the current research, the DEGs in both sets were significantly enriched in ABC transporters. In the HJNP_HJSP gene set, there were 36 DEGs encoding proteins related to ABC transporters, namely, 9 *CoABCB*, 21 *CoABCC*, and 6 *CoABCG* genes. *CoABCC10* was the major gene upregulated in HJSP, while the 6 *CoABCG* genes were downregulated in HJ after self-pollination. In the HSNP_HSSP gene set, there were 24 DEG-encoding proteins related to ABC transporters, namely, 6 *CoABCB* and 18 *CoABCC* genes. All 24 DEGs were significantly upregulated, but seventeen DEGs were significantly upregulated in both the two groups, namely, 6 *CoABCB* and 11 *CoABCC* (Figure 4C and Supplementary Table S2). The results indicated that *CoABCB* and *CoABCC* might be associated with the LSI of Camellia oil trees.

### 2.7. DEGs Related to the Phosphatidylinositol Signaling System

External stimulation can accelerate the metabolic activity of plant membrane lipids. Lnositol phospholipid compounds on the cell membrane are important secondary messengers in plant signal transduction. The current study revealed 7 DEGs (6 upregulated and 1 downregulated genes) in the HJNP_HJSP gene set and 10 DEGs (8 upregulated and 2 downregulated genes) in the HSNP_HSSP gene set, which were involved in the phosphatidylinositol signaling system (Figure 4B and Supplementary Table S3). In the HJNP_HJSP gene set, the phosphatidylinositol 4-phosphate 5-kinase 6-like (*PIP5K6*), diacylglycerol kinase 1-like isoform X2 (*DGK1*), and phosphoinositide phospholipase C 6-like (*PLC6*) genes were upregulated in the HJNP_HJSP gene set, while the diacylglycerol kinase 2 (*DGK2*) gene was downregulated. In the HSNP_HSSP gene set, the 1-phosphatidylinositol-3-phosphate 5-kinase (*PIP5K6*), FAB1B-like isoform X1 (*FAB1B*), inositol-pentakisphosphate 2-kinase-like isoform X2 (*IPK1*), inositol hexakisphosphate, diphosphoinositol-pentakisphosphate kinase VIP2-like (*VIP2*), and *PLC6* genes were upregulated in HSSP compared with HSNP, while the phosphoinositide phospholipase C 2-like *(PLC2*) gene was downregulated. Comparing the two groups, we found that there were more types of DEGs in the HSSP group, and *PLC6* was upregulated in both HSSP and HJSP.

### 2.8. DEGs Related to ROS and the Ca^2+^ Signaling Pathway

Numerous studies have shown that ROS are essential for signal transduction in the SI of Papaver and Prunus avium as well as in pollen tube polar development [35,36]. When SI occurred, ROS levels in the pollen tube increased rapidly for the gene set [37], six *colRBOH* genes, three glutathione S-transferase (*colGST*) genes and three glutathione peroxidase (*colGPX2*) genes, all of which were involved in ROS scavenging, and six receptor-like protein kinase FERONIA (*colFER*) genes were upregulated in HJSP compared with HJNP. In the HSNP_HSSP gene set, four respiratory burst oxidase protein-like (*RBOH*) genes, one glutathione S-transferase U7-like (*GSTU7*) gene, two mitochondrial alternative oxidase 1d (*AOX1A*) genes and five receptor-like protein kinase HERK 1 isoform (*FER*) genes were upregulated. Overall, the results obtained indicated that ROS may be crucial in the LSI of camellia oil tree and that the abovementioned DEGs may be significant in balancing ROS production and scavenging.

Ca^2+^, a secondary messenger, is crucial for controlling pollen germination and preventing self-fertilization, and several DEGs related to Ca^2+^ signaling genes were identified in this study. Calcium-dependent protein kinase (*CDPK*) is an important primary sensory receptor for calcium signaling and is located at the top of pollen tubes [37]. One predicted *CDPK* has a high expression level in HJSP. Ca^2+^ outflow requires the *ACA* and *CCX*, which can provide energy. Five calcium-transporting ATPase (*ACA*) genes and five cation/calcium exchanger 1-like (*CCX*) genes were upregulated in the HJNP_HJSP gene set. Similarly, four calmodulin-binding protein (*CAM*)-encoding genes were upregulated in the HSNP_HSSP gene set (Figure 4A,B).

### 2.9. A Putative S-RNase Gene Involved in SI of C. oleifera

S-RNase-mediated SI widely exists in Solanaceae, Scrophulariaceae, and Rosaceae species [38,39], and it is a significant and widespread mechanism for promoting cross-pollination and avoiding self-fertilization in plants. S-RNase belongs to the RNase T2 family, and based on the number of in-trons and evolutionary connections, these sorts of genes may be categorized into three groups: S-like RNases include groups I and II, while S-RNases are belong to group III [40]. In this study, a DEG encoding extracellular ribonuclease LE-like isoform X1 (*RNS1*), which is highly homologous to RNase genes, was most highly expressed in the HSNP_HSSP gene set.

### 2.10. Identification and Characterization of T2 RNases in Camellia Oil Tree

**Figure 5:**
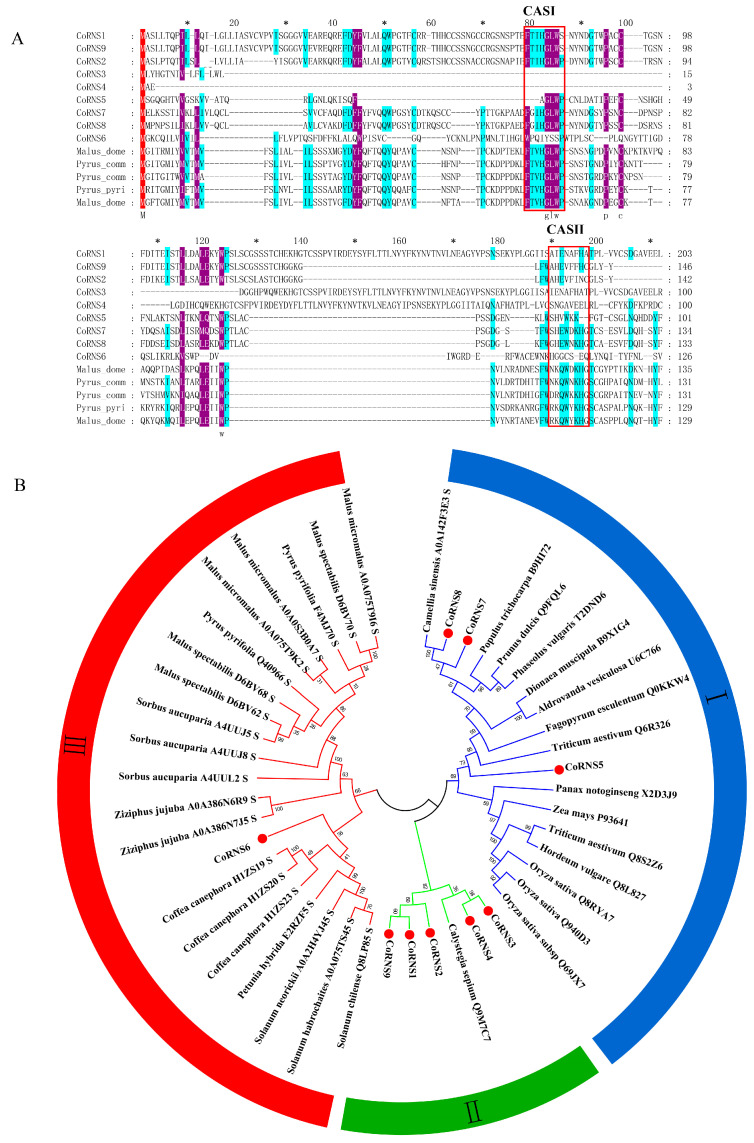
Analysis of the 9 RNase T2 genes from *Camellia oleifera*. (**A**) An alignment of the amino acids in 9 RNase T2 genes and the S-RNase from other species, every 20 amino acids were marked with *, the two conserved active site (CAS) regions are indicated with a red box. (**B**) Phylogenetic analysis of the 9 Nase T2 genes.

In the current study, a total of 9 RNase T2 genes were identified in *C. oleifera*, named *CoRNS1*, *CoRNS2*, *CoRNS3*, *CoRNS4*, *CoRNS5 CoRNS6*, *CoRNS7*, *CoRNS8*, and *CoRNS9*. The length of the coding DNA sequence (CDS) varied from 429 to 789 bp, and there were between 143 and 315 amino acids. The typical molecular weight of RNase T2 enzymes usually ranges from 25 to 32 kDa [41]. The molecular weights of 5 of the proteins encoded by the aforementioned gene fell within this range, whereas those of the proteins encoded by CoRNS2, CoRNS3, CoRNS4, and CoRNS9 fell outside of that range (15.65~17.39 kDa). The theoretical pI of the proteins encoded by these 9 genes varied from 4.94~8.77. These hypothetical RNase T2 proteins have two basic and six acidic pI values. The signal peptide of the candidate RNase proteins in the pistil of *C. oleifera* was predicted by an online website (http://www.cbs.dtu.dk/services/SignalP-4.1/ (accessed on 3 December 2021)), and the results showed that most RNase T2 proteins, except for *CoRNS4* and *CoRNS5*, contained signal peptides (Table 2). Multiple alignment of the amino acid sequences of the nine proteins and five other S-RNases clearly revealed similarities with Rosaceae species (Figure 5A). The results indicated that *CoRNS1*, *CoRNS6*, *CoRNS7*, and *CoRNS8* contained conserved active sites named CAS I and CAS II, whose RNase T2 protein function depends on its histidine residues [42], while *CoRNS1* and *CoRNS6* have only one active site.

According to the biological function of the RNases, we categorized RNase T2s into the S-RNase and S-like RNase categories [43]. S-RNases are specifically expressed in flowering plants to regulate plant SI, and S-like RNases have a major role in responses to biotic and abiotic stress, such as antimicrobial defense, phosphate scavenging, the enhancement of stress tolerance and tRNA cleavage [44]. S-RNases and S-like RNases can also be divided into three groups: class I, class II, and class III. S-like RNases correspond to classes I and II, and S-RNases correspond to class III [42]. A phylogenetic tree was constructed using the sequences of our 9 RNase T2 proteins, as well as those of 15 identified S-like RNases and 21 S-RNase proteins that were pulled at random from the protein database to better understand the precise categorization of the RNase T2 genes in the Camellia oil tree (http://www.uniprot.org (accessed on 3 December 2021)). The tree was built using the neighbor-joining (NJ) method in MEGA 7.0 and contained monocotyledonous plant species including rice, wheat, and maize as well as dicotyledonous plant species, such as apple, pear, and coffee. According to the characteristics of their protein structures, all of the sequences were grouped into three groups, as shown in Figure 5B, and *Co*RNase6 clustered on the same small branch as the S-RNase from coffee. This small branch clustered with a large branch consisting of annotated S-RNases from Rosaceae and Solanaceae. Another branch contained *CoRNase1*, *CoRNase2*, *CoRNase3*, *CoRNase4*, *CoRNase5*, *CoRNase9* and 14 other S-like RNases that were identified. Interestingly, *CoRNase7* and *CoRNase8* were closely related to an S-RNase from *C. sinensis*, and *CoRNase7* was identified as a DEG in the transcriptome of HSSP compared with HSNP. In addition, compared to S-RNases, the proteins on the small branch had a closer relationship with S-like RNases (Figure 5B). Taken together, these results indicated that *CoRNase6*, *CoRNase7*, and *CoRNase8* were likely involved in SI, but the biological functions of the 9 proteins need to be further studied.

### 2.11. Prokaryotic Expression Analysis of RNases

**Figure 6:**
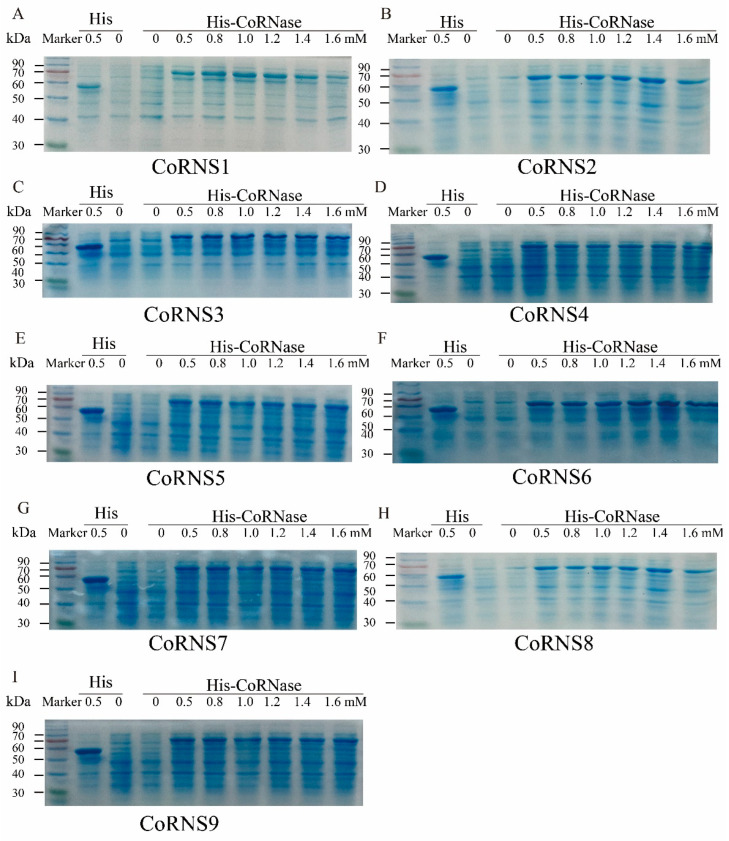
Protein expression analysis of the RNase T2 genes. (**A**) Small amount of induced expression of His-*Co*RNS1 fusion protein. (**B**) Small amount of induced expression of His-*Co*RNS2 fusion protein. (**C**) Small amount of induced expression of His-*Co*RNS3 fusion protein. (**D**) Small amount of induced expression of His-*Co*RNS4 fusion protein. (**E**) Small amount of induced expression of His-*Co*RNS5 fusion protein. (**F**) Small amount of induced expression of His-*Co*RNS6 fusion protein. (**G**) Small amount of induced expression of His-*Co*RNS7 fusion protein. (**H**) Small amount of induced expression of His-*Co*RNS8 fusion protein. (**I**) Small amount of induced expression of His-*Co*RNS9 fusion protein.

Based on the principle of homologous recombination, 9 His-*Co*RNase recombinant expression vectors corresponding to the 9 RNase T2 genes were constructed and transformed into *Escherichia coli* Rosetta (DE3) for the induction and expression of recombinant proteins. The 9 His-*Co*RNases were successfully induced in response to isopropyl β-D-1-thiogalactopyranoside (IPTG) at concentrations of 0, 0.5, 0.8, 1.0, 1.2, 1.4 and 1.6 mmol/L. The resulting protein sizes ranged from 73~90 kDa. These results indicated that the His-*Co*RNase prokaryotic expression vectors were successfully constructed and that the target proteins were successfully expressed in response to the induction of IPTG (Figure 6). With increasing IPTG concentration, the protein expression increased slightly or did not change to a large degree.

### 2.12. Subcellular Localization of RNases

**Figure 7:**
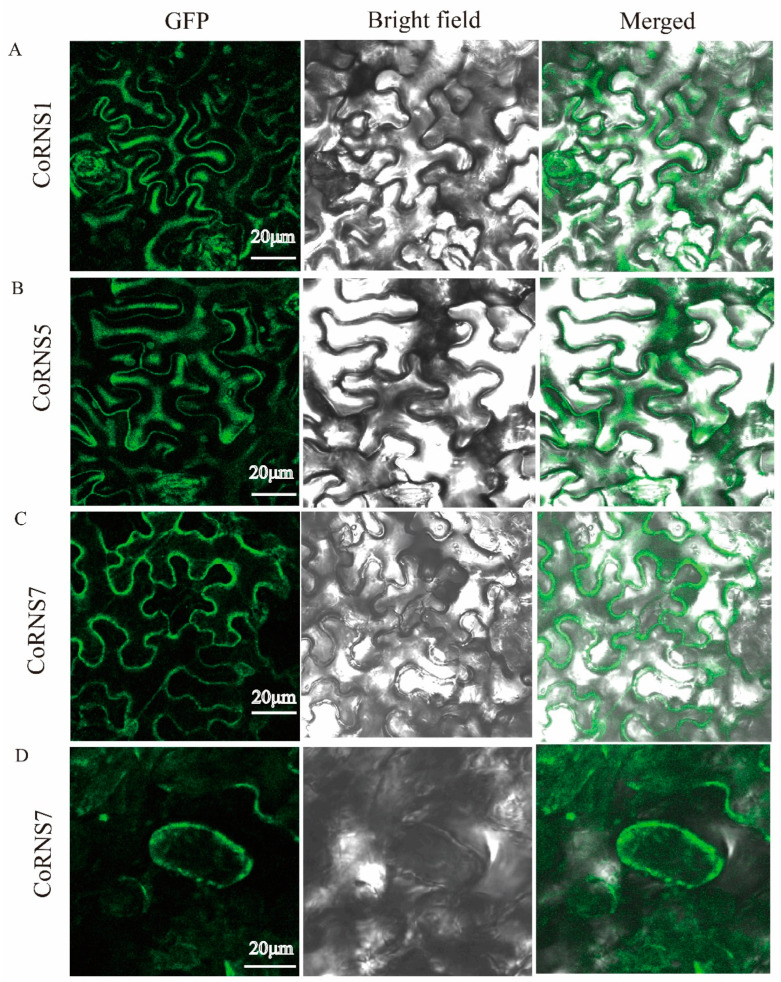
The subcellular localization of CoRNS1, CoRNS5, and CoRNS7. (**A**) Subcellular localization analysis of *Co*RNS1 fusion protein in leaf epidermal cells of *Nicotiana benthamiana.* (**B**) Subcellular localization analysis of *Co*RNS5 fusion protein in leaf epidermal cells of *Nicotiana benthamiana.* (**C**) Subcellular localization analysis of *Co*RNS7 fusion protein in leaf epidermal cells of *Nicotiana benthamiana.* (**D**) Subcellular localization analysis of *Co*RNS7 fusion protein in leaf epidermal cells of *Nicotiana benthamiana* after treatment with NaCl solution.

According to predictions from online sites (http://www.csbio.sjtu.edu.cn/bioinf/plant-multi/ (accessed on 4 December 2021)), *CoRNase8* from the Camellia oil tree was predicted to be localized in the cytosol, *CoRNase5* was predicted to be localized in the nucleus, and the resident RNase localized to the extracellular space. To verify this, the *CoRNase1*, *CoRNase5* and *CoRNase7* were fused to green fluorescent protein (GFP), and the three GFP-fusion proteins were temporarily expressed in *Nicotiana benthamiana* leaves, as illustrated in Figure 7. The green fluorescence of *CoRNase1* and *CoRNase5* was visible in the cytoplasm, and the green fluorescence of *CoRNase7* was exclusively restricted to the plasma membrane. After treatment with NaCl solution, the tobacco cytoplasm was obviously separated from the cell wall, and the green fluorescence became concentrated (Figure 7). Taken together, these results indicated that *CoRNase1* and *CoRNase5* were cytoplasmic proteins, while *CoRNase7* was a plasma membrane-localized protein.

### 2.13. Quantitative Real-Time PCR (qRT–PCR) Validation

**Figure 8:**
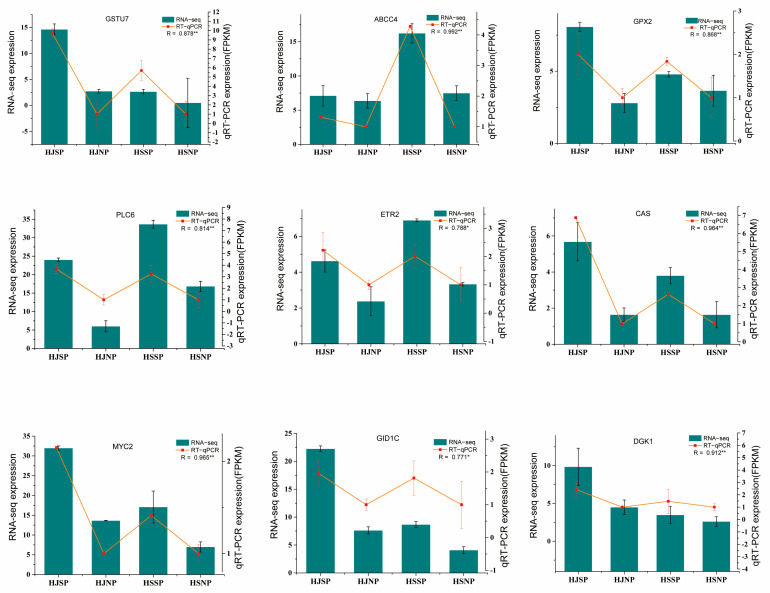
Quantitative qRT-PCR validation of 9 DEGs identified related to the LSI in transcriptome. The R2 represents the Pearson coefficient between the qRT-PCR and RNA-seq results and asterisks indicate significant differences (* *p* < 0.05; ** *p* < 0.01).

To assess the credibility of the RNA-seq data, 9 DEGs involved in SI during SP and NP were chosen as targets based on their expression fold changes and FPKM, and *CoGAPDH* was chosen as an internal control and verified via qRT–PCR. Data from qRT-PCR and RNA-seq were then compared. As Figure 8 shows, the change trend of these DEGs in the four samples according to the qRT–PCR results was highly similar to that of the transcriptomic data sets. This means that the data obtained from RNA-seq were reliable.

## 3. Discussion

Previous studies found that phytohormones, such as auxin (IAA), ethylene (ET), CKs, GA, brassinosteroids (BRs), ABA, salicylic acid (SA), and JA, are small signaling molecules that are crucial in regulating plant endogenous developmental processes and responses to environmental stress [45,46,47]. Numerous investigations have revealed that phytohormones play vital roles in plant SI [48,49]. In this study, the ovaries of self-pollinated *C. oleifera* HJ and HS showed a significant enrichment of DEGs in plant hormone signal transduction pathways. Earlier studies discovered that soon after pollination, the content of endogenous GA in the pistils of pears increased, but when the pollen tube ceased growing due to self-incompatibility, the concentration of endogenous GA in the pistil was reduced, as was also shown in apples [50]. In this study, we found that *PIF3* and *GID1*, which positively regulate the GA response in both cultivars of *C. oleifera*, were upregulated in the two comparison groups. JA is crucial to the plant defensive response. Gu [51] found that S-RNase in pollen tubes could promote an increase in JA content in self-pollinated flowers. Here, we found that *MYC2*, a transcription factor with positive regulatory functions in the JA signaling pathway, was upregulated in both groups. ABA is crucial for the plant’s responses to stress, flower bud induction, seed dormancy and germination [52,53,54], and it can induce the synthesis of S-RNase involved in pear SI [33]. As a negative regulator of self-pollination, *PYR/PYL* was downregulated in the ovaries of *C. oleifera* HJ. These results corresponded to those of earlier reports on cacao, whose ABA components were increased in flowers after self-pollination [55], which suggested that the ABA signaling cascade participated in the pistil response to pollen. Taken together, the findings show that the plant hormone signaling pathway participates in the LSI reaction of *C. oleifera*.

ABC transporters can transport multiple types of substrates across the plasma membrane via ATP hydrolysis in response to environmental stress [56,57]. A previous study in *M. domestica* demonstrated that *MdABCF* in pollen could combine with S-RNase, a key protein of GSI, and transport it to the cytoplasm of the pollen tube to facilitate its cellular cytotoxic function [34]. In the present study, DEGs were significantly enriched in ABC transporters. Twenty-six DEGs, namely, 6 *CoABCB*s and 20 *CoABCC*s, were highly expressed in the HJNP_HJSP gene set. Similarly, 22 DEGs, namely, 4 *CoABCB*s and 18 *CoABCC*s, were highly expressed in the HSNP_HSSP gene set, and 14 were commonly upregulated. Overall, these findings show that *CoABCB* and *CoABCC* participate in the LSI of *C. oleifera*.

The phosphatidylinositol signaling system is among the most universal molecular reaction mechanisms involved in signal transduction. The inositol phosphate compounds IP3 and DAG on the cell membrane are secondary messengers in plant signal transduction pathways [58]. Phospholipase C (PLC) also plays an important role in phosphatidylinositol (4,5) P_2_ hydrolysis, and the downstream product phosphatidic acid (PA) can mitigate PbrS-RNase cytotoxicity to protect the pollen tube [26]. In the current study, the phosphatidylinositol signaling system showed significant enrichment in DEGs in the ovary transcriptome during SI, among which the *PLC6* gene was upregulated in both the HJNP_HJSP gene set and the HSNP_HSSP gene set. We assume that overexpression of *PLC6* might contribute to the hydrolysis of phosphatidylinositol and trigger complex protective mechanisms against environmental stimuli and harm.

When they are present in excessive amounts, ROS have been described as toxic metabolic products [59,60,61]. Many studies have confirmed that ROS participate in flower development and many pollen-related processes, such as the growth process of the pollen tube apex [62], stigma and pollen recognition and SI [63]. In the current study, we found 19 DEGs that were involved in ROS metabolism in the HSSP_HSNP gene set and 16 DEGs in the HJNP_HJSP gene set. These DEGs included *RBOH*, *GPX2*, *FER*, *GST*, and *GSTU7*. In addition, RBOH during both self- and nonpollination was upregulated. We previously found that the content of ROS in plants showed a significant upward trend at 12–48 h after self-pollination and peaked at 48 h, and respiratory burst oxidases (*RBOHs*) were found to be essential in ROS generation [60]. This means that ROS function in plant defense during this time. Meanwhile, Ca^2+^ also plays an vital role in pollen tube growth and SI [64,65]. It was reported that Ca^2+^ can promote the production of ROS during pollen tube tip growth [35] (Figure 5A). Ca^2+^ signaling cascades could lead to PCD in Papaveraceae, which is part of the GSI mechanism [66]. In the current study, we found that three CaM genes had considerably higher levels of expression in the two groups. Taken together, these results indicated that ROS and Ca^2+^ signaling-related genes play vital roles in the LSI reaction of *C. oleifera*.

S-RNases have been demonstrated to be female determinant genes involved in the rejection of self-produced pollen and causing GSI in plants of the Solanaceae, Scrophulariaceae and Rosaceae families [67,68,69]. The Camellia oil tree was identified as undergoing LSI according to the location at which pollen tube growth was inhibited instead of the genetic mechanism, and the molecular mechanism of LSI is not yet clear. In the current study, a putative S-RNase gene was found to be significantly upregulated in the HSNP_HSSP gene set, and another eight genes highly homologous to T2 RNase were cloned. Prokaryotic expression analysis indicated that these 9 proteins were successfully expressed, and subcellular localization indicated that *CoRNase1* and *CoRNase5* were cytoplasmic proteins. Zhang [10] found an S-RNase gene that was significant highly expressed in a style that was self-pollinated vs. one that was cross-pollinated in tea (*C. sinensis* L.), which is in the same family as the Camellia oil tree (Theaceae family). Both were under LSI, as identified by the pollen tubes being inhibited in the ovaries after self-pollination [70]. Taken together, these results indicate that the LSI of Camellia oil trees might be based on GSI.

## 4. Materials and Methods

### 4.1. Plant Materials, Pollination Treatment and Sample Collection

Two SI camellia oil tree varieties, Huajin (HJ) and Huashuo (HS), were selected in this study. The two cultivars (no voucher specimen) were selected for a comparative regional assessment out of 84 *C. oleifera* clones, and these two varieties are the main promoted varieties in the cultivation of *Camellia oleifera*. They are grown at the Huju Forest Farm in Zhuzhou city, Hunan Province. HJ reached its peak blooming toward the end of October, while HS was at the beginning of December. These two varieties grew normally and produced a typical number of flowers.

The anthers were harvested from mature blossom buds of two cultivars, HJ and HS, and put it at room temperature of 25 °C for 8 h. Once the pollen was scattered, the pollen was collected. Four pollination treatments were designed: HJ × HJ (HJSP), HS × HS (HSSP), non-pollination of HJ (HJNP) and non-pollination of HS (HSNP). On sunny days in late October and early December, pollination was performed from 8:00–11:00 am and from 1:00–5:00 pm. In accordance with the four pollination techniques, the blossom buds were emasculated and pollinated. Then all treated materials were wrapped in a sulfate paper bag. The ovaries for RNA-seq and qRT-PCR were collected 48 h after pollination and kept at 80 °C (3 biological duplicates), with each replicate containing 50 ovaries. Pistils were collected for pollen tube observations at various time intervals (0, 12, 24, 36, 48, 60, 72, 84 h) and then fixed with Carnoy’s fixative [71].

### 4.2. Data Surveys and Cytological Observations

The pistils were extracted from Carnoy’s fixative and then softened by being macerated in sodium hypochlorite. After they were incubated for 2 h at room temperature, they were washed with deionized water and macerated in 8 M NaOH for two hours. After being cut into three to five portions along the vertical axis using a medical blade, the styles were stained with aniline blue for six hours. Fluorescence microscopy was then used to observe the pollen tube development.

The survey of fruit setting rate was conducted approximately sixty days after pollination, and more than 300 flowers were investigated for three times each time.

### 4.3. RNA-Seq, Transcriptome Assembly and Functional Annotations

Total RNA was extracted using the TRIzol reagent (Invitrogen, Carlsbad, CA, USA). A Ribo-ZeroTM M agnetic Kit (Epicenter, Madison, WI, USA) was then used to extract the rRNA. Then the enriched mRNA was fragmented into short fragments using NEBNext^®^ Magnesium RNA Fragmentation Module (NEB#E6150S, New England Biolabs, Ipswich, MA, USA) for 5 min at 94 °C and reverse transcribed into cDNA with random primers. Using DNA polymerase I, dNTPs, RNase H, and buffer, second-strand cDNA was created. After that, the cDNA fragments were purified using a QIAquick PCR extraction kit from Qiagen in Venlo, the Netherlands. After that, they were polyadenylated, end repaired, polyadenylated, and ligated to Illumina sequencing adapters. With the use of agarose gel electrophoresis, PCR, and an Illumina NovaSeq 6000 (Gene Denovo Biotechnology Co., Guangzhou, China), the ligation products were submitted to size selection for sequencing.

Raw reads collected by the sequencing machines, which could influence the subsequent analysis and assembly. The reads were further filtered using fastp [72] to provide high-quality clean reads, and rRNA sequences were then eliminated using alignment. *Camellia oleifera* Huashuo genomic data have been acquired (this study has not yet been published.). Using HISAT 2.2.4 [73] and the default settings for “-rna-strandness RF” and other parameters, paired-end clean reads were mapped to the reference genome using an index of the reference genome. StringTie v1.3.1 [74,75] was used in a reference-based manner to build the mapped reads of each sample. Four databases, namely, the NR, Swiss-Prot, GO, and KEGG databases, were used for analyze the unannotated transcripts. DESeq2 was used for identify the DEGs [76] software between the two different groups (and by edgeR [77] between two samples).

### 4.4. Gene Cloning, Multiple Sequence Alignment and Phylogenetic Analysis

Based on the transcriptomic data, the full-length CDSs of 9 RNase T2 family genes were obtained. Total RNA was extracted from *C. oleifera* pistils according to the instructions of the E.Z.N.A. ^®^Gel Extraction kit. cDNA reverse transcription was carried out using R212, Hiscript ^®^III 1st Strand cDNA Synthesis Kit, which was made by Vazyme, China. Primer Premier 5.0 was used to design the corresponding 9 pairs of primers, which Shanghai Shenggong Bioengineering Co., (Shanghai, China) synthesized. The 9 RNase T2 genes were cloned using the Panta ^®^ Max Surper-Fiderlity DNA Polymerase Kit P505 produced by Vazyme, China. Target gene fragments were detected via gel electrophoresis and recovered by a gel recovery kit. The recovered target fragments were subsequently ligated into a pUC19 vector, which was then transformed into *E. coli* DH5α. Bacteria exhibiting good growth were selected and sequenced. With minimal deviation, all tests were carried out in accordance with each kit’s instructions.

Vector NTI programs were used to align and blast the amino acid sequences of the 9 RNase T2 genes. To divide these 9 RNase T2 genes into two categories, S-RNases and S-like RNases, the neighbor-joining (NJ) method of MEGA 7.0 was used to create a phylogenetic tree. The phylogenetic tree contained 9 candidate RNase proteins of Camellia oil tree and 36 RNase T2 enzymes reported for different species. The information was obtained from the UniProt database (http://www.uniprot.org/ (accessed on 3 December 2021)).

### 4.5. Recombinant Protein Expression

To analyze the subcellular localization of the 9 CoRNase genes, these 9 RNase T2 genes were cloned into the pCAMBIA1300-35S-GFP vector for subcellular localization using CloneExpress ^®^ II One Step Cloning Kit C112, which was produced by Vazyme, China. Transforming the recombinant vector into Agrobacterium by electric shock, injecting it into the lower epidermis of tobacco for transient expression with osmotic buffer. Incubate the tobacco injected with bacterial solution in dark for 4–8 h, and then move it to normal conditions for 2–3 days, Observation of subcellular localization using laser confocal microscopyby LSM 510 laser confocal microscopy. GFP was detected at 488 nm for excitation, and it was released at 495–545 nm for emission.

To ensure that these 9 CoRNases could be successfully expressed as proteins and for subsequent experiments, these 9 RNase T2 genes were cloned into the pCold-TF vector to create His-tagged recombinant protein using CloneExpress^®^ II One Step Cloning Kit C112, The TF-His tag has a molecular weight of approximately 48 kDa. Inducing the production of proteins in E. coli Rosetta (DE3) cells. Recombinant DE3 cells were cultured at 37 °C overnight with the proper antibiotics. The CoRNases protein expression was induced with IPTG at different concentrations (0, 0.5, 0.8, 1.0, 1.2, 1.4, and 1.6 mM), and the culture medium was shaken at 15 °C for 24 h to facilitate the expression of the recombinant protein. Finally, the proteins in the sample were detected by SDS-PAGE electrophoresis.

### 4.6. qRT–PCR Verification

A total of nine DEGs associated with the LSI of the Camellia oil tree were chosen and assessed by qRT-PCR in order to confirm the correctness of the RNA-seq results. A plant RNA kit (OMEGA, New York, NY, USA) was used to extract the RNA from those samples, and HiScript^®^ II Q RT Su-perMix for qPCR (+gDNA wiper) (Vazyme, Nanjing, China) was used to reverse transcribe and remove the potential remaining gDNA. By using Primer Premier 5.0, specific primers for the 9 DEGs were designed (Supplementary Table S4). The ChamQ Universal SYBR qPCR Master Mix #Q711 (Vazyme, Nanjing, China) was used in the QRT-PCR procedure. CoGAPDH was selected as the reference gene for the Camellia oil tree in this experiment, which was carried out on a CFX96 Real Time PCR System (Bio-Rad, Hercules, CA, USA). Using the 2^−∆∆CT^ method, the target gene’s expression level was determined. Three duplicates of each sample were examined.

## 5. Conclusions

In this study, cytological observations, transcriptome, and RNase T2 family analyses were carried out to reveal the molecular mechanism of LSI in Camellia oil tree. We found that the degree of SI among different Camellia oil tree cultivars were significantly different, and the self-fruiting rate in Huashuo (34.20%) was significantly higher than that in Huajin (19.14%). Based on the analysis of the transcriptome, plant hormone signal transduction, ATP-binding cassette (ABC) transporters, the phosphatidylinositol signaling system, reactive oxygen species (ROS) metabolism, and Ca^2+^ signaling were mainly involved in the LSI of Camellia oil tree. In addition, we found a putative S-RNase gene that was involved in LSI of Camellia oil tree, and other 8 RNase T2 genes were identified and analyzed. The results should help elucidate the mechanism of LSI in Camellia oil trees, and the discovery of T2 RNases provided evidence that Camellia oil trees might be under RNase-based gametophytic SI.

## Figures and Tables

**Table 1:** Details of sequence reads and unigenes from de novo assembly for 12 RNA samples.

Sample	Raw_Reads (Mb)	Clean_Reads (Mb)	Clean_Bases (Gb)	Q20	Q30	Unique_Map	Multi_Map	Total_Map
HJNP-1	66.78	66.50	9.92	98.00%	94.16%	39,267,036 (59.08%)	21,089,363 (31.73%)	60,356,399 (90.81%)
HJNP-2	69.22	69.02	10.10	98.10%	94.41%	40,222,163 (58.31%)	21,918,936 (31.78%)	62,141,099 (90.09%)
HJNP-3	61.92	61.71	9.02	97.87%	93.82%	36,100,019 (58.54%)	19,422,333 (31.50%)	55,522,352 (90.04%)
HJSP-1	47.98	47.89	7.15	98.33%	94.74%	28,096,446 (58.71%)	15,250,400 (31.87%)	43,346,846 (90.57%)
HJSP-2	50.43	50.33	7.52	98.15%	94.26%	29,748,589 (59.14%)	15,790,304 (31.39%)	45,538,893 (90.53%)
HJSP-3	52.69	52.60	7.86	98.07%	94.04%	30,664,191 (58.34%)	16,592,787 (31.57%)	47,256,978 (89.90%)
HSNP-1	61.12	60.95	9.09	97.94%	93.91%	38,592,078 (63.37%)	18,130,734 (29.77%)	56,722,812 (93.14%)
HSNP-2	62.36	62.18	9.27	97.77%	93.62%	39,226,394 (63.13%)	18,632,072 (29.98%)	57,858,466 (93.11%)
HSNP-3	72.42	72.21	10.76	97.95%	94.07%	44,810,287 (62.10%)	22,744,249 (31.52%)	67,554,536 (93.62%)
HSSP-1	44.88	44.80	6.70	98.28%	94.59%	28,700,957 (64.11%)	13,046,307 (29.14%)	41,747,264 (93.25%)
HSSP-2	61.69	61.58	9.21	98.12%	94.19%	39,366,886 (63.97%)	17,537,945 (28.50%)	56,904,831 (92.47%)
HSSP-3	57.18	57.09	8.64	98.26%	94.52%	36,229,624 (63.51%)	16,581,192 (29.07%)	52,810,816 (92.58%)

**Table 2:** Characteristics of RNase T2 genes in *Camellia oleifera* ‘Huashuo’.

Name	ID	Length of CDS	Amino Acid	Molecular Weight	PI	Signal Peptide
*Co*RNS1	oil_tea_GLEAN_10114714	748	315	34.82	5.6	yes
*Co*RNS2	oil_tea_GLEAN_10391745	429	142	15.65	6.02	yes
*Co*RNS3	oil_tea_GLEAN_10141428	480	159	17.75	5.39	yes
*Co*RNS4	oil_tea_GLEAN_10391744	448	143	16.0	4.94	no
*Co*RNS5	oil_tea_GLEAN_10004004	480	159	17.39	8.6	no
*Co*RNS6	oil_tea_GLEAN_10401437	753	250	28.39	8.77	yes
*Co*RNS7	oil_tea_GLEAN_10185682	684	227	24.97	4.59	yes
*Co*RNS8	oil_tea_GLEAN_10400090	717	262	26.54	5.42	yes
*Co*RNS9	oil_tea_GLEAN_10340465	457	146	16.1	5.78	yes

## Data Availability

All data generated or analyzed during this study were included in this published article and the additional files. The RNA-seq data can be found in SRA data library under accession number PRJNA867027. https://dataview.ncbi.nlm.nih.gov/object/PRJNA867027?reviewer=a52f1c6obqs98rp0uio5a346db (accessed on 12 May 2022).

## Supplementary Materials

The following supporting information can be downloaded at: https://www.mdpi.com/article/10.3390/plants12101932/s1, Table S1: The unigenes fail to be mapped to the reference genome of Camellia oil tree in transcriptome; Table S2: Expression profiles of genes in ABC transporters in transcriptome; Table S3: Expression profiles of genes in phosphatidylinositol signaling system in transcriptome; Table S4: Primers used in qRT-PCR.
